# Removal and extraction efficiency of Quaternary ammonium herbicides paraquat (PQ) from aqueous solution by ketoenol–pyrazole receptor functionalized silica hybrid adsorbent (SiNPz)

**DOI:** 10.1186/s13065-019-0599-2

**Published:** 2019-07-09

**Authors:** Shehdeh Jodeh, Ghadir Hanbali, Said Tighadouini, Smaail Radi, Othman Hamed, Diana Jodeh

**Affiliations:** 1 Department of Chemistry, An-Najah National University, P. O. Box 7, Nablus, Palestine; 2 LCAE, Department of Chemistry, Faculty of Sciences, Mohamed Premier University, 60000 Oujda, Morocco; 3 LCAE, Faculté des Sciences, Université Mohamed I, 60000 Oujda, Morocco; 4 Division of Plastic and Reconstructive Surgery, Johns Hopkins All Children’s Hospital, St. Petersburg, FL USA

**Keywords:** Ketoenol–pyrazole receptor, Adsorption, Paraquat, Kinetics, Isotherm

## Abstract

Pesticides and herbicides have been used extensively in agricultural practices to control pests and increase crop yields. Paraquat (PQT^2+^, 1,1-dimethyl-4,4-dipyridinium chloride) is one of the herbicide that belois classified as bipyridines and is used over the world. The objective of this study is to use ketoenol–pyrazole receptor functionalized silica hybrid as adsorbent for removal PQT^2+^ from aqueous solution. The adsorbent was synthesized, and characterized using scanning electron microscopy (SEM), nuclear magnetic resonance (NMR), Thermal analysis and other techniques. Different experimental parameters such as the effect of the amount of adsorbent, solution pH and temperatures and contact times were studied. Pseudo-order kinetics models were studied, and our data followed a pseudo second order. Experimental data were analyzed for both Langmuir and Freundlich models and the data fitted well with the Langmuir isotherm model. To understand the mechanism of adsorption, thermodynamic parameters like standard enthalpy, standard Gibbs free energy, and standard entropy were studied. The study indicated that the process is spontaneous, exothermic in nature and follow physisorption mechanisms. The novelty of this study showed surface of pyrazol-enol-imine-substituted silica (SiNPz) has the ability to highlight the surface designed for efficient removal of PQT^2+^, from aqueous solutions more than other studies. The study also showed that ketoenol–pyrazole receptor can be regenerated in five cycles using HNO_3_ without affecting its adsorption capacity.

## Introduction

Pesticides have been used in agriculture to overcome pests and increase crop yields. They are used to reduce weeds, insecticides and fungicides. The amount of these pesticides that needed are not well known and most of the farmers exceeded the required quantity [1]. Most industries and food processing companies are always releasing some pesticides through their effluents [2]. Pesticides are organic compounds and they affect the environment in different ways. There are different types of pesticides categories including organophosphates, carbamates, substituted urea compounds, organochlorines, and pyrethroids. Due to their dangerous effect and toxicity in the environment, different research areas are involved to get rid of them from the environment [3].

Lately, agricultural types in Palestine are aiming to avoid low plant development and increase the production and the quality of the products. These changes help to introduce higher levels of herbicides in the agricultural ecosystem [4]. The main output of these agricultural practices is the contamination of soils and waters, which leads to degrade the soil–water–plant system and bioaccumulate herbicide residues.

Paraquat (PQT^2+^, 1,1-dimethyl-4,4-dipyridinium chloride) is a herbicide and belongs to the class of the bipyridines. It is one of the most widely used herbicides in the world and forbidden in some countries. The advantages of paraquat over other herbicides is very quick and non-selective action to kill green plant tissue upon usage [5].

In the last years, several studies have been given to PQT^2+^, mainly due to the high rate of poisoning and fatalities attributed to it [4].

Several studies have been carried out for the removal of PQT^2+^ from aqueous medium and wastewater. One of them is related to the oxidation of PQT^2+^, which emphasize the destruction of the structure of the pesticide [5]. In this study, several reagents can be used for this study and can be enhanced by applying ultraviolet radiation, which increases the formation of free radicals. Some disadvantages of this experiment are the production of toxic substances if the degradation process was not carried right [6–8].

Another method of removal of PQT^2+^ is adsorption on solid adsorbents using different substrates and nanomaterials. Various adsorbents such as activated carbon [9], biological tissues [10] and modified materials [11] have been employed for the adsorption of PQT^2+^ from aqueous solutions. In previous studies, sawdust and peanut shell powder were explored as adsorbents for the removal of phosphorus and other dyes from aqueous solutions [12–16].

Pesticides and herbicides are determined using instrumentation such as gas chromatography (GC) and high-performance liquid chromatography (HPLC) [17]. Their degradation is involved by crops through their metabolites [18]. There are several methods that are used for determination of pesticides like nanotechnology-based protocols were used to investigate these problems [19]. Some examples like, some metals and silica nanoparticles are used for such studies [19]. In this study, the ability of pyrazole and its derivatives to play as ligands with sp^2^ hybrid nitrogen donors have been the study areas of several scientists. This is shown in different research and published papers in this field [20, 21]. Besides that, ketoenol moiety has an important type of ligand in view of its distinct structural characteristics and high synthetic utility [17]. Research on β-ketoenol derivatives and their metal complexes have been studied by a number of phenomena’s such as their important practical application.

This kind of molecules have two possibilities of coordination sites and can act as a uni- or bidentate ligand or coordinate to the metal atom through monoionic or neutral form. Sometimes they form a bridge between two metal atoms. It is obvious that will lead for the possibility to opens this kind of ligand to be grafted onto silica gel and increase adsorption capacity toward heavy metals or other contaminates of interest.

The goal of this study is to report the investigation of the fabrication of highly branched adsorbent and chelated material using covalent immobilization of a prepared mixed ligand (β-ketoenol–pyrazole) onto silica particles to study the adsorption of PQT^2+^ from aqueous solutions.

## Experimental

### Materials and methods

The solvents and chemicals used in this study were purchased from Aldrich, USA. All of them with high purity. Silica gel (E. Merch) with a particle size in the range of 70–230 mesh, median pore diameter 60 Å, was activated using heat at 160–170 °C within 24 h. The salivating agent 3-aminopropyltrimethoxysilane (Janssen Chimica) was very pure. All the characterization of the samples was described and reported in our previous study for the removal of heavy metals [17]. Paraquat dichloride was purchased from (Fluka, Steinheim, Germany).

### Synthesis of (2*Z*)-1-(1,5-dimethyl-1*H*-pyrazole-3-yl)-3-hydroxybut-2-en-1-one

As we reported in previous study [22], amount of ethyl 1,5-dimethyl-1*H*-pyrazole-3-carboxylate (30 mmol) dissolved in 30 mL of toluene and added to a suspension of sodium (52.5 mmol) in 50 mL of anhydrous toluene. Acetone (2.5 g; 42.5 mmol) dissolved in 10 mL of toluene was added at very low temperature. The final solution was shacked vigorously at room temperature for 48 h.

**Scheme 1:**
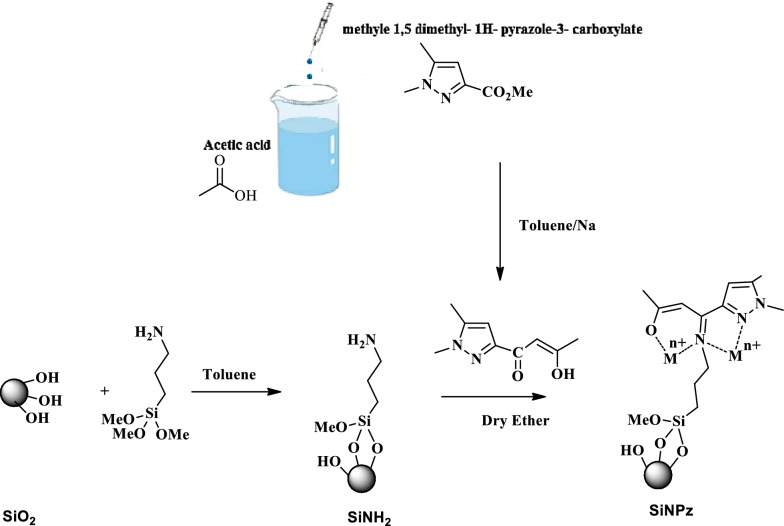
The synthesis mechanism of modified chelating compounds

The precipitate was filtered and washed several times with toluene and then dissolved in water. The final pH was close to 5. The solution was extracted with CH_2_Cl_2_ and the bottom layer (organic) was dried using anhydrous sodium sulfate and all solvents were evaporated to have very concentrated sample using vacuum. The compound (2*Z*)-1-(1,5-dimethyl-1*H*-pyrazol-3-yl)-3-hydroxybut-2-en-1-one (Scheme 1) was obtained from the residue which was chromatographed on silica using CH_2_Cl_2_ as eluant. The final product was characterized by X-ray crystallography and NMR as described in our previous study [22, 23].

### Synthesis of 3-aminopropylsilica (SiNH2)

To accomplish this synthesis, reaction between the silylating agent and silanol groups on the silica surface was occured. An amount of activated silica gel SiO_2_ (30 g) mixed with about 150 mL of dried toluene was refluxed and stirred under nitrogen atmosphere for about 2 h. After that, 10 mL of aminopropyltrimethoxysilane was added dropwise to the suspended solution and the final mixture was refluxed for 2 days. The precipitate was filtered and washed several times with both toluene and ethanol. The solution was extracted using a mixture of ethanol and dichloromethane (1:1) for about 12 h to separate all residues (Scheme 1). In this stage we named the immobilized silica gel SiNH2 which was dried at room temperature [22].

### Synthesis of pyrazol-enol-imine-substituted silica (SiNPz)

To prepare and synthesizedf SiNPz, amount of 3-aminopropylsilica (SiNH2) (5 g) and (2*Z*)-1-(1,5-dimethyl-1*H*-pyrazol-3-yl)-3-hydroxybut-2-en-1-one (3 g) were dissolved in 60 mL of dry diethyl ether. The mixture was stirred mechanically for 24 h at room temperature. As mentioned before, the solution was filtered and Soxhlet extracted using acetonitrile, methanol and dichloromethane for 12 h, respectively. Final product was dried at 70 °C for 24 h.

### Sample characterization

#### Elemental analysis

The elemental analysis for the synthesis of SiNH2 showed 4.46% of carbon and 1.66% of nitrogen. While the synthesis of SiNPz showed 9.73% of carbon and 2.8% of nitrogen. The variation of carbon and nitrogen between the two samples indicating the variation of organic moieties. The increase of both nitrogen and carbon in the second sample (SiNPz) indicated that the (2*Z*)-1-(1,5-dimethyl-1*H*-pyrazol-3-yl)-3-hydroxybut-2-en-1-one was attached to SiNH2.

#### Surface properties

All NMR, FT-IR, SEM, surface pore volume and thermal analysis were done for the sample prepared with SiNH2 and SiNPz and the sample was used for the application of studying the efficiency of removing heavy metals from aqueous solution [22].

#### Measurements of PQ in water

In our study for determination PQ in solution, a sensitive method was used and reported by Rai et al. [23, 24]. Where sodium borohydride is used as reducing reagent for the reduction of PQ to form a stable blue colored free radical ion. The advantages of the method are simple, reproducible, nontoxic reducing agent and excellent stability of the blue free radical ion.

In summary, 1000 mg L^−1^ -aqueous solution of paraquat (PQT) was prepared by dissolving 69.1 mg of paraquat dichloride (Aldrich, USA) in deionized water to make 50 mL of solution in a volumetric flask. Different working standard solutions and calibration curves were prepared by appropriate dilution from the stock solution depending on the experiment.

The absorption spectra of the blue colored solution showed maximum absorbance at 600 nm while the reagent blank had a negligible absorbance at this wavelength.

The reproducibility of the method was studied by replicate analysis of 3.0 µg of PQ in 10 mL solution for 5 days. The SD and relative SD of absorbance values were found to be ± 0.0053 and 1.47% respectively.

#### Adsorption kinetics

The adsorption kinetics experiments were studied as follow: (50 mg/L, 100 mg adsorbant and agitation speed of 300 rpm). The studies on the adsorption using the SiNPz adsorbent have indicated that the adsorption showed very fast and increased slowly after 50 min up 200 min. The samples were drawn from the beaker by a pipet of 10 mL at different interval times of 1, 5, 10, 30, 60, 90, and 180 min. Each sample was filtered with filter paper of 45 µm and analyzed using the spectrophotometer (Hitachi UV-1500A) at 600 nm. Both the effect of temperatures (15, 25, 35 and 45 °C) and the pH 2, 4, 6, 10 and 12) were studied. Each time we study one parameter we keep the other constant. This experiment was done with repletion of 3 times and the average was used when we analyzed the data.

#### Adsorption isotherm

In each experiment, about 100 mg of SiNPz adsorbent was placed into a shaker bath at 25 ± 0.1 °C and initial pH of 11.0 for all experiments. Isotherm experiments were handled by shaking (at 300 rpm) with a known volume (50 mL) of paraquat solutions at different initial concentration and specified contact time. The concentration of paraquat was analyzed at the end of each contact period and the measurements were repeated 3 times.

## Results and discussion

The parameters affecting the adsorption of paraquat, such as dosage, initial concentration, pH, and temperature, were studied. In our study, for those parameters, we kept all variables constant except the one we want to study.

## Investigation of adsorption parameters

### pH effect on PQT^2+^ adsorption

**Fig. 1:**
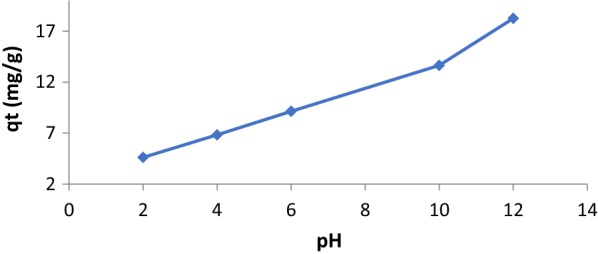
Effect of pH on PQT^2+^ removal

The amount of paraquat adsorption increases with pH (Fig. 1). As usual, dsorption depends on the type and morphology of the adsorbent surface. By decreasing pH, the H^+^ usually competes with adsorbate at different exchange sites in the system.

From (Fig. 1), the amount of adsorption was very small at pH = 2 and increased when the solution become basic (as pH increases).

The small amount of adsorption at low pH (< 2) was due to the high mobility of H^+^ and adsorbed over PQT^2+^ and this lead to the increased of the adsorbed amount of cationic paraquat which was increased in response to the increasing number of negatively charged sites that exist due to the loss of H^+^ from the surface [25].

### Temperature effect on PQT^2+^ adsorption

**Fig. 2:**
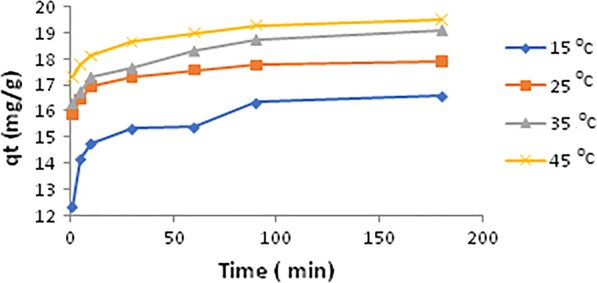
Effect of temperature on PQT^2+^ removal by SiNPz

The effect of temperature on the adsorption equilibrium is shown in Fig. 2. From which it can be seen that the adsorption capacity was favored by increasing temperature. The capacity towards the adsorption of PQT^2+^ increased 1.2-times when the temperature was increased from 15 to 45 °C and the temperatures chosen is very close to that find in drinking water [26, 27]. This was proven in our results when we studied the thermodynamics parameters and it was endothermic. This suggest that adsorbate has very high affinity for this pesticide and there is no competition for the solvent which leads to formation of monolayer of PQT^2+^ covering the adsorbate surface.

### Concentrations effects on PQT^2+^ adsorption

**Fig. 3:**
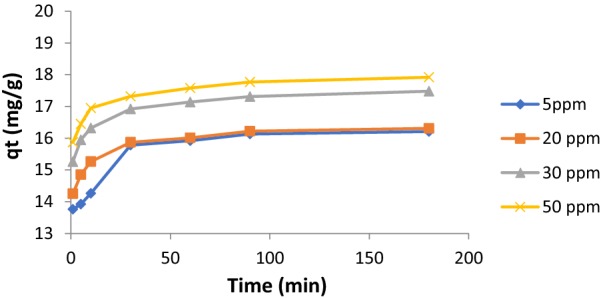
Effect of concentration on PQT^2+^ removal by SiNPz

Effect of initial concentration of PQT^2+^ adsorption processes was studied with fixing previous conditions. The results are shown in Fig. 3. The figure shows the effect of contact time on the removal of paraquat by SiNPz as a function of the amount removed (qt). The figure showed that the amount removed for paraquat pollutants increased during the first 15 min and reached equilibrium after that. When concentration increases from 5 to 50 mg/L, the adsorption capacity is also increasing. This may be because of a gradual increase in the electrostatic attraction between PQT^2+^ and the absorbent desired active sites [28].

### Adsorption isotherm

To understand the adsorption capacity, we have to design an experiment at a specific temperature to remove paraquat from the aqueous solution.

There are several isotherm models like Langmuir, Freundlich, BET, etc. which can be applied at all temperatures. All of these models have equations that can be used, and the data will be fit into these equations. One of the factors that can lead to the type of isotherm model is the correlation coefficients, R^2^ [28].

The Langmuir equation is one of the most used and can be expressed as [29]:

1 $$\frac{{C_{e} }}{{q_{e} }} = \frac{1}{{bQ_{o} }} + \frac{1}{{Q_{o} }}C_{e}$$

where Ce represents the equilibrium concentration of the adsorbate (mg/L); b is usually, the Langmuir affinity constant (L/mg). Q_o_ is the adsorption capacity at equilibrium (mg/g); and q_e_ is the amount of adsorbate per unit mass of adsorbent (mg/g).

The other type of isotherm model is Freundlich isotherm is an empirical formula which used for low concentrations and can be presented as [30]:

2 $$\log q_{e} = \log K_{F} + \frac{1}{n}\log C_{e}$$

where K_F_ is the Freundlich constant that deal with adsorption capacity (mg/g) and n is the heterogeneity coefficient which leads to how favorable the adsorption process (g/L).

**Fig. 4:**
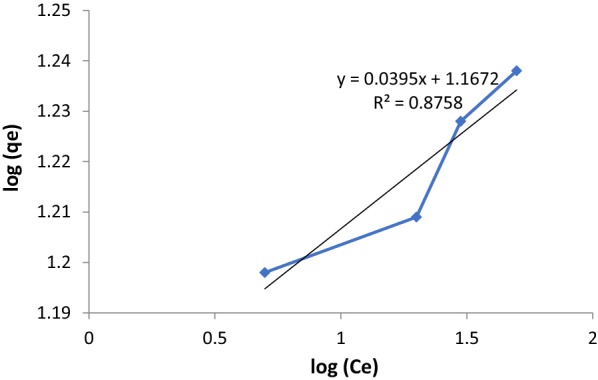
Isothermal adsorption of paraquat in aqueous solution onto SiNPz at 298 K of Freundlich model

**Fig. 5:**
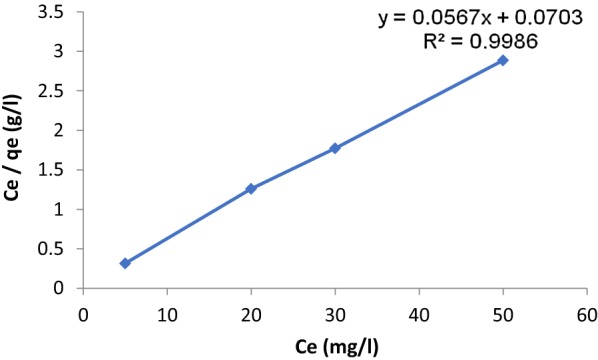
Isothermal adsorption of paraquat in aqueous solution onto SiNPz at 298 K of Langmuir model

In the above equation the slope 1/n, having the value between 0 and 1, which describe adsorption intensity and surface heterogeneity, If the value of 1/n is close to zero, this means more heterogeneous [31], and if the value of 1/n less than one this indicates Langmuir-type isotherm and at the same time becomes difficult to adsorb additional adsorbate molecules at higher adsorbate concentrations [32]. Table 1 and Figs. 4 and 5 summarizes the whole data of Freundlich and Langmuir isotherms, indicating the satisfactorily good correlation between the model and the experimental data. The Langmuir isotherm shows very well fit to the data, with correlation coefficients (R^2^) of 0.9986 compared with 0.7070 for Freundlich isotherm. A value for 1/n (0.0393) below one leads to a Langmuir-type isotherm. It is observed that the monolayer adsorption capacity (i.e., q_m_) and Langmuir constant (i.e., K_L_), are high enough and very closed to other previous studies [32]. This result is reasonable since the adsorption affinity and monolayer adsorption capacity will be enhanced by the increase in surface area observed for the adsorbent. Therefore, the monolayer adsorption capacities of adsorbents are mainly dependent upon physical properties such as Brunauer–Emmett–Teller BET surface area.

**Table 1:** Parameters in Langmuir and Freundlich adsorption isotherm models of paraquat onto ketoenol–pyrazole at 298 K

Langmuir isotherm parameters	Q_o_ (mg/g)	b (L/mg)	R^2^
	17.63	0.80	0. 9986
Freundlich isotherm parameters	n	1/n	R^2^
	25.44	0.0393	0.707

### Adsorption kinetics

Presenting the experimental data through kinetics equations like the Lagergren pseudo-first-order model, the pseudo-second-order model will describe the mechanism of adsorption and degradation of paraquat in aqueous solution. Such studies give information about the possible mechanism of adsorption of paraquat and different transition states on the final complex of paraquat and the adsorbent. From the reactions parameters like rate constants and adsorption capacity factors, one can have an idea about the adsorption dynamics and this will help the industry for other applications.

The adsorption experimental data of paraquat by the SiNPz were analyzed using the most common kinetic models to understand the nature of the adsorption process.

**Fig. 6:**
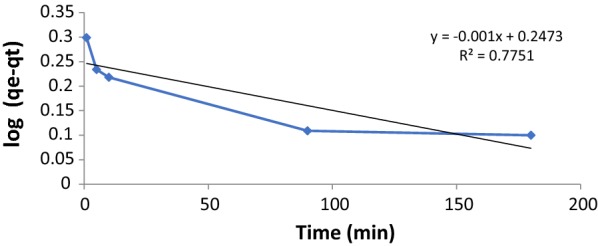
Pseudo-first order plots for the adsorption of paraquat by SiNPz (experimental conditions: 10 mL sample volume, 100 mg SiNPz, and paraquat concentration 20.0 mg L^−1^)

The adsorption of paraquat by solid adsorbents such as SiNPz was fitted to one of the most used kinetic models; Lagergren pseudo-first-order model [33], the equation can be written as the following:

3 $$\log \left( {q_{e} - q_{t} } \right) = \log q_{e} - \frac{{K_{1} }}{2.303}t$$

where k_1_ (min^−1^) is the pseudo-first-order adsorption rate coefficient, and q_e_ and q_t_ are the values of the amount adsorbed per unit mass at equilibrium at time t, respectively. Plotting ln (q_e_ − q_t_) vs. t for paraquat did not give straight lines as it is clear from Fig. 6 with very low regression coefficients (0.707) as shown in Table 2.

**Table 2:** Pseudo first order and pseudo second order kinetic model parameters for PQT^2+^ adsorption by SiNPz

Pseudo first order	q_e_ (exp) 17.32	k_1_	q_e_ (cal)	R^2^	K _id_
		0.002	0.753	0.7751	0.156
Pseudo second order		k_2_	q_e_ (cal)	R^2^	C
		0.096	17.95	0.9897	16.153

From Table 2, the calculated values of the amount adsorbed at equilibrium (q_e_, calc) were far from the experimental values (q_e_, exp) for the pseudo -first order which means that the adsorption cannot be represented by this model.

The pseudo-second-order equation was also used for describing the adsorption of the paraquat by SiNPz [34].

The equation of the pseudo-second-order rate is given as:

4 $${\text{t}}/{\text{q}}_{\text{t}} = { 1}/{\text{K}}_{ 2} \left( {{\text{q}}_{\text{e}} } \right) 2 { } + {\text{ t}}/{\text{q}}_{\text{e}}$$

**Fig. 7:**
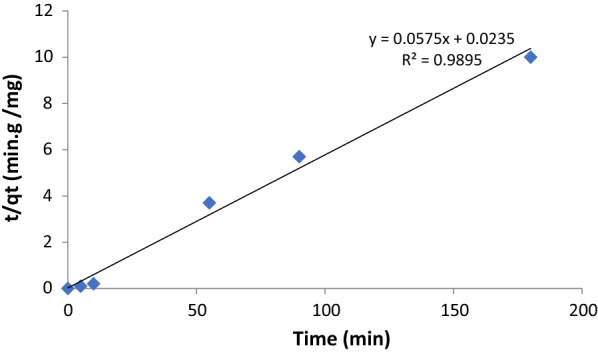
Pseudo-second-order plots for the adsorption of paraquat by SiNPz

[Experimental conditions: 10 mL sample volume, 100 mg ketoenol–pyrazole, and paraquat concentration 20.0 mg L^−1^), where k_2_ (g/(mg min)] is the pseudo-second-order rate coefficient, and q_e_ and qt are the values of the amount adsorbed per unit mass at equilibrium and at any time t, respectively. From Fig. 7 and Table 2, the pseudo-second-order rate equation to the adsorption of the paraquat by SiNPz showed good converging for the experimental data, and excellent regression coefficients (R^2^ = 0.9897)

In case of pseudo-second order, it is clear from Table 2, that both the correlation coefficient R^2^ which is very close to one and the values for both (q_e_ Calc) and (q_e_ Exp) were very close and this indicates that the adsorption followed pseudo -second order.

#### Adsorption rate-controlling mechanism

The sorption of paraquat by SiNPz is a very complex process where both characteristics of both (adsorbate and adsorbent) plays an important role. Different factors will be involved in this process: bulk solution will be involved when adsorbate diffused from the solution to the boundary surface of the solution surrounding SiNPz. Other phenomena are film diffusion when paraquat diffuse through the film surrounding SiNPz. Finally, what we called pore diffusion when paraquat finds pores inside SiNPz. Usually, the slowest one will control the adsorption.

**Fig. 8:**
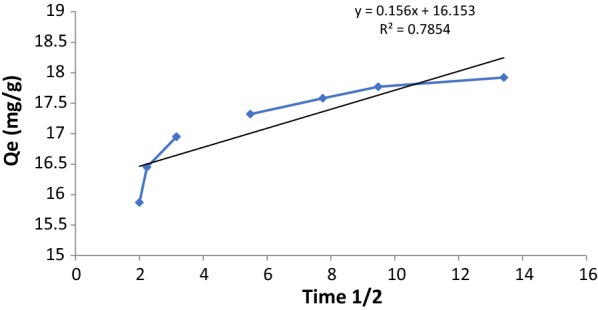
Intra-particle diffusion model plots for the adsorption of paraquat by SiNPz (experimental conditions: 10 mL sample volume, 100 mg SiNPz, and 20.0 mg L^−1^)

Webber and Morris developed an equation describing the intraparticle diffusion and can be written as the following equation [35].

5 $${\text{q}}_{\text{t}} = {\text{ K}}_{\text{id}} {\text{t}}^{ 1/ 2} + {\text{ C}}$$

where q_t_ (mg g^−1^) is adsorption capacity at any time (t), k_id_ (mg g^−1^min^1/2^) is the intra-particle diffusion rate constant, and C (mg ^g−1^) is a constant proportional to the thickness of the boundary layer. Usually, the larger value of C, the better and greater boundary layer thickness. Plotting data of q_t_ against t^1/2^ usually describe the process of diffusion controlled. From the plot, if there are multiple linear plots, means the adsorption of paraquat by SiNPz is controlled by more than one step. Figure 8, represent the experimental data paraquat adsorption by SiNPz using Webber–Morris model and the data showed two straight lines. Usually, the first portion of the straight line represents the diffusion process which is controlled by the external surface of the adsorbent, while the second one represents the intraparticle diffusion. The intercepts of the straight lines usually, gives the boundary layer thickness. In our study, we have two steps which means that the diffusion was controlled by the external surfaces and intraparticle diffusion. Another thing, the data did not pass through origin indicating a difference in diffusion rates between the two steps as shown in Table 2.

### Thermodynamic studies

In this study, different parameters were calculated like the standard free energy, standard enthalpy, and standard entropy. The aim of this study is to understand spontaneity and to understand the nature of adsorption. The following equation was used [36]:

$${\text{D }} = {\text{ q}}_{\text{e}} /{\text{C}}_{\text{e}}$$

where q_e_ is the amount of paraquat adsorbed by SiNPz, (mg/g) at equilibrium, and C_e_ is the equilibrium concentration of paraquat in the solution (mg/L). The ΔH and ΔS can be calculated from the following equation [37]:

$${\text{Ln D }} = \Delta {\text{S}}/{\text{R }}{-} \, \Delta {\text{H}}/{\text{RT}}.$$

**Fig. 9:**
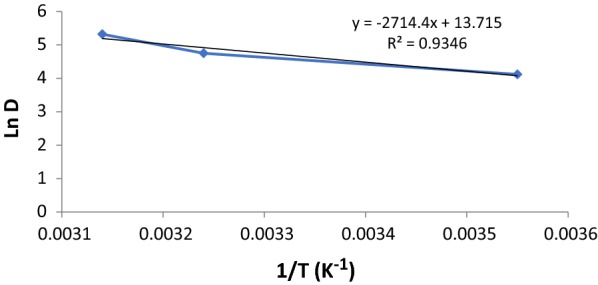
A plot of ln D vs. 1/T for the calculations of thermodynamic parameters for the adsorption of paraquat by SiNPz (experimental conditions: 50 mL sample volume, 50 mg SiNPz, and concentration 20.0 mg L^−1^)

Plotting ln D vs. 1/T for the adsorption of paraquat, a straight line was obtained and shown in Fig. 9 and Table 3.

**Table 3:** The values of the calculated thermodynamic parameters of PQT^2+^ adsorption

ΔS° (J/mol k)	ΔH° (kJ/mol)	ΔG° (kJ/mol)			
		288 K	298 K	308 K	318 K
0.114	22.56	− 10.27	− 11.41	− 12.55	− 13.69

The standard free energy ΔG° can be calculated using this equation:

6 $$\Delta {\text{G}}^\circ = \Delta {\text{H}}^\circ - {\text{ T}}\Delta {\text{S}}^\circ .$$

From Fig. 9, both ΔH and ΔS can be calculated from the slope and the intercept of the straight line. The ΔH values was +22.56 kJ/mol, for the adsorption of paraquat by SiNPz from the aqueous solution. This positive value indicates the endothermic nature of the adsorption of paraquat by SiNPz, which confirmed our previous study of the effect of temperature that adsorption increased when temperature increased. Also, the value of ΔH suggests a strong affinity between paraquat by SiNPz and the physical nature of the adsorption. The low value of ΔS, 0.114 J/mol K, suggested very low of randomness at the SiNPz/solution interface during the adsorption and immobilization of paraquat.

The ΔG values were negative which indicates that the adsorption of paraquat by SiNPz was favored and spontaneous. The negative values of ΔG, and positive values of ΔH, and ΔS suggested that the adsorption of paraquat process is an entropy-driven process.

#### Regeneration of adsorbent

In order to make the adsorption more environmentally friendly, regeneration experiment was studied. Regeneration is an important factor to determine the cost-effectiveness and the possibility of reuse several times. The main important factor is the possibility to reuse paraquat that has been adsorbed for other things.

Thermodynamics study showed that the adsorption process is governed by physisorption, indicating a week force between adsorbent and adsorbate. This means that the regeneration process is feasible. Also, from the study of pH effect on adsorption, the removal efficiency increased as pH increased. In this case, decreasing pH will enhance the desorption process. This suggests that washing the contaminated ketoenol pyrazole with an acid like HNO_3_ is more efficient than with basic solution.

**Fig. 10:**
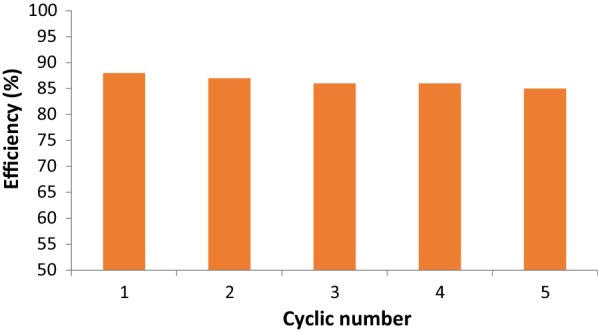
Adsorption–desorption experiments of paraquat by SiNPz

Figure 10 shows the cycles of adsorption–desorption experiments using 6 mM HNO_3_ and reused for 5 successive removal processes with efficiency higher than 87%. This reasonable result is due to the fact that entropy is usually occurring from the bulk solution like adsorbent’s pores to more dilute HNO_3_ solution. This means that adsorption of paraquat is reversible, and bonding between active sites is not strong.

## Conclusion

Pesticides have been used extensively in agriculture to control pests and increase crop yields. They are used to control weeds, insecticides and fungicides. This study approved that SiNPz receptor could be used as an adsorbent for paraquat from aqueous solution in a short time with high removal efficiency. The optimization parameter for adsorption like, pH, temperature, and dosage were studied and found to playan important role in the capacity of adsorption increased with increasing temperatures and pH. The adsorption isotherm was studied, and the data were best fitted with the Langmuir model. The data were fitted to both pseudo–first order and pseudo-second order and the results fitted much better to pseudo-second order using both correlation coefficient R^2^ and q_e_ experiment was very closed to the calculated one. Thermodynamics study showed that the adsorption is spontaneous and exothermic with physisorption nature of the adsorption process.

The regeneration studies confirmed that the adsorbent can be reused for several times with adsorption capacity of more than 87%. This means that the adsorption process is efficient simple and cost-effective and can be used in large-scale industry.

## Data Availability

The datasets used and/or analyzed during the current study are available from the corresponding author on reasonable request.

## Abbreviations

Paraquat: PQT^2+^, 1,1-dimethyl-4,4-dipyridinium chloride
SEM: scanning electron microscopy
NMR: nuclear magnetic resonance
SiNPz: pyrazol-enol-imine-substituted silica
GC: gas chromatography
HPLC: high-performance liquid chromatography
SiNH2: 3-aminopropylsilica
R^2^: correlation coefficients
Ce: equilibrium concentration
b: Langmuir affinity constant
Q_0_: adsorption capacity at equilibrium (mg/g)
q_e_: amount of adsorbate per unit mass of adsorbent (mg/g)
K_F_: Freundlich constant
n: heterogeneity coefficient
K_L_: Langmuir constant
BET: Brunauer–Emmett–Teller
K2: pseudo-second-order rate coefficient, the amount adsorbed per unit mass at equilibrium and at any time q_e_ and q_t_
Kid: intra-particle diffusion rate constant
C (mg ^g−1^): a constant proportional to the thickness of the boundary layer
ΔH: Enthalpy
ΔS: Entropy
ΔG°: standard free energy
R (8.314 J/K.mol): ideal gas constant
